# Women Empowerment and Its Relationship With Anxiety and Depression Symptoms Among Currently Married Women in Bangladesh: A Nationally Representative Cross‐Sectional Study

**DOI:** 10.1155/da/7941286

**Published:** 2026-10-31

**Authors:** Md. Mosfequr Rahman, Md. Ashik Ulla Ashik, Raufun Hasan Arnob, Shamima Akter, Mahmudul Hasan Tonmoy, Md. Golam Mostofa

**Affiliations:** ^1^ Department of Population Science and Human Resource Development University of Rajshahi Rajshahi Bangladesh ru.ac.bd

**Keywords:** anxiety, attitude toward violence, decision-making, depression, social independence, SWPER, women’s empowerment

## Abstract

**Background:**

Mental disorders are prevalent, with women experiencing a higher incidence of mental health issues compared to men. The disparity in mental disorders may be attributed to women’s disadvantaged social status. Data linking women’s empowerment to mental health are limited. This study analyzes the relationship among currently married women in Bangladesh.

**Method:**

A sample of married women (*n* = 15,813) was obtained from the 2022 Bangladesh Demographic Health Survey (BDHS). The survey‐based women’s empowerment (SWPER) index was used to assess women’s empowerment. The evaluation of anxiety and depression symptoms was conducted using the generalized anxiety disorder (GAD‐7) scale and the Patient Health Questionnaire (PHQ‐9).

**Results:**

Attitudes toward violence and social independence were correlated with reduced adjusted scores on the GAD‐7 (β‐coefficients: −0.34 for attitudes toward violence and −0.16 for social independence) and PHQ‐9 scales (−0.24 for attitudes toward violence and −0.13 for the social independence domain). However, the decision‐making domain showed a positive association. Results also show that a unit increase in the standard deviation (SD) of the attitude toward violence score was associated with higher odds of moderate to severe anxiety symptoms (odds ratio [OR]: 0.79; 95% confidence interval [CI]: 0.68–0.91) and depression symptoms (OR: 0.80; 95% CI: 0.69–0.94). The social independence domain correlates with moderate to severe depressive symptoms, exhibiting lower odds (OR: 0.86; 95% CI: 0.75–0.99).

**Conclusion:**

This study identified associations between various dimensions of women’s empowerment and mental health issues among women, highlighting the necessity of prioritizing women’s empowerment within mental health initiatives in Bangladesh and similar contexts.

## 1. Introduction

Women’s empowerment denotes the attainment of enabling resources that may augment their agency or ability to make strategic life choices to promote their welfare, within contexts of evolving constraints [1, 2]. The empowerment of women serves as a crucial mechanism for attaining global sustainable development, focusing on enhancing women’s self‐worth, their capacity for autonomous decision‐making, their entitlement to govern their lives both domestically and externally, and their right to shape social change [3]. There is increasing interest in research and policy to identify strategies for enhancing women’s empowerment. Empowering women is a significant objective in its own right and can also lead to various positive outcomes for women and children. A substantial body of research indicates that women who experience greater empowerment, as defined by various criteria, achieve improved health care outcomes [4–7].

Women in low‐ and middle‐income countries exhibit a heightened vulnerability to prevalent mental health disorders in comparison to men, a susceptibility that can be ascribed to a multifaceted interaction of biological, social, and environmental influences [8–10]. Nevertheless, the disproportionate impact of mental health challenges faced by women in South Asian societies may surpass that observed in other regions globally [11, 12]. Conventional gender roles and practices in South Asia frequently undermine the status of women, diminish their allocation of household resources, and restrict their access to education, employment, and avenues for cultivating social capital [13–16]. Beyond the weight of psychological distress, the mental health challenges faced by women carry significant implications for the well‐being of children. In low‐income mothers residing in low‐ and middle‐income nations, there exists a correlation between depression and adverse social, health, and cognitive outcomes in their children, notably including malnutrition, stunting, and cognitive delays [17, 18].

The impact of women’s empowerment on their subjective well‐being or mental health is still poorly understood worldwide, particularly in Bangladesh, despite conflicting results from recent global studies [19]. Cross‐sectional analysis of empirical data utilizing different definitions of empowerment and mental health has revealed a positive correlation between them [20–25]. However, in a study conducted in Thailand, multivariate analysis revealed no significant association between decision‐making power and depressive symptoms [26]. Multiple channels exist through which empowerment may influence well‐being, and these channels typically indicate a positive relationship, albeit not consistently. Empowered women often experience increased social support and greater access to economic resources, both of which are associated with improved mental health outcomes [26–28].

Mental disorders are prevalent in Bangladesh, impacting approximately one‐fifth of adults and one‐eighth of children and adolescents, with women exhibiting greater susceptibility to mental illness than men [29–32]. Despite the significant prevalence of mental problems in Bangladesh, mental health issues remain mostly unacknowledged and insufficiently researched [33]. Disparities and inequalities persist despite the Government of Bangladesh’s dedication to attaining gender equality and empowering women, a goal consistent with the Sustainable Development Goals (SDGs) initiated in 2015. Therefore, this paper seeks to elucidate the relationship between women’s empowerment, as quantified by the survey‐based women’s empowerment (SWPER) index, and women’s mental health issues, assessed through the generalized anxiety disorder (GAD‐7) scale and the Patient Health Questionnaire (PHQ‐9), within a large nationally representative sample from Bangladesh. We posited that women’s empowerment is inversely associated with anxiety and depressive symptoms.

Noteworthy metrics for assessing gender equality encompass the Gender Development Index, the Gender Gap Index, and the Gender Inequality Index. These indices assess countries based on the level of women’s equality relative to men. Nevertheless, they are deficient in their capacity for subnational or subgroup analysis. In response to this deficiency, a study conducted in Africa presented the SWPER index [34], which provides a more thorough evaluation of the influence of women’s empowerment on health interventions and outcomes. In contrast to other indices, SWPER facilitates both intracountry and intercountry comparisons as well as temporal trend analysis. Utilizing the comprehensive SWPER empowerment metrics, alongside extensive socioeconomic data and several mental health indicators, we offer a distinctly nuanced analysis of how diverse aspects of empowerment influence well‐being in this context. The study’s significance is emphasized in several aspects. This study addresses a gap in the literature in Bangladesh since it is the first investigation into the correlation between women’s empowerment and mental disorders in the country. Second, a substantial sample from a nationally representative survey was employed to fulfill the study objective.

## 2. Method

### 2.1. Data

We acquired data from the latest, ninth nationally representative cross‐sectional dataset, from the 2022 Bangladesh Demographic Health Survey (BDHS). The BDHS gathered data on various socioeconomic, demographic, women’s empowerment, and mental health‐related factors concerning women aged 15–49 years through a two‐stage stratified cluster sampling approach. Initially, 675 enumeration areas (EAs: 237 in urban settings and 438 in rural settings) were chosen as primary sampling units (PSUs) based on a probability proportional to the size of the EA. Subsequently, a systematic sample of 45 households per EA was randomly selected. The survey was effectively conducted across 674 clusters following the removal of a rural cluster in Cox’s Bazar, Chittagong, owing to security issues. In each EA, 30 out of 45 households were randomly chosen for the comprehensive individual questionnaire, which was given to all eligible women in those households. In the other 15 households, a condensed version of the questionnaire was administered to all eligible women. In the households that were interviewed, a total of 30,358 ever‐married women aged 15–49 were recognized as suitable for individual interviews. Out of these, 20,217 were deemed eligible for the full questionnaire, while 10,141 qualified for the short questionnaire. In total, interviews were conducted with 30,078 women, resulting in an outstanding response rate of 99% across the entire sample. Women who met the criteria for the complete questionnaire were included in this study. Out of 30,078 eligible women aged 15–49 years interviewed, 10,091 were excluded for not being interviewed in the mental health survey, 1000 were excluded for not being currently married, and 3174 (16.7%) were excluded due to missing responses in women’s empowerment variables. Consequently, the final analytical sample for this study comprised 15,813 currently married women. The comprehensive methodology can be found elsewhere [35]. Ethical approval was not necessary for this study, as we utilized a publicly available secondary dataset.

### 2.2. Outcomes

Anxiety and depression, two common mental health disorders among women, were assessed using two different indices. The 7‐item GAD‐7 was used to evaluate anxiety symptoms [36]. Second, the PHQ‐9 was used as a depression symptom assessment tool [37]. Both scales focus on symptoms experienced in the 2 weeks preceding the survey. The severity of symptoms for both the GAD‐7 and PHQ‐9 scales is represented by a Likert scale, with scores of 0, 1, 2, and 3 corresponding to the response options: not at all (never), several days (rarely), more than half the days (often), and nearly every day (always). In the 2022 BDHS, the validated Water Glass Pictorial Scale was used to assess symptom intensity. An empty glass means “never,” a one‐fourth‐filled glass means “rarely,” a half‐filled glass means “often,” and a three‐fourths‐filled glass means “always.” This graphic chart helped describe respondents’ feelings and contextualize symptom intensity [35].

The GAD‐7 score varies from 0 to 21, with recommended thresholds indicating minimal anxiety at 0–4, mild at 5–9, moderate at 10–14, and severe at 15–21 [36]. The PHQ‐9 score varies from 0 to 27, where a higher score suggests an increased probability of major depressive disorder. The proposed thresholds for categorizing depression severity are as follows: minimal 0–4, mild 5–9, moderate 10–14, moderately severe 15–19, and severe 20–27 [37]. This sample’s GAD‐7 and PHQ‐9 Cronbach’s alphas values are 0.77 and 0.85, respectively, indicating adequate internal consistency.

In our analysis of the GAD‐7 and PHQ‐9 scales, we used continuous variables to quantify the severity of anxiety and depressive symptoms. Additionally, for secondary analysis, we incorporated a binary variable for each scale, adhering to the established threshold for moderate to severe anxiety and depression, defined as a symptom score of 10 or higher on the respective scales.

### 2.3. Exposures

We assessed women’s empowerment through SWPER Global, an individual‐level metric designed for women aged 15–49 years who are married or in a union. This metric is a refined iteration of the SWPER initially developed for African countries [38]. A comprehensive exposition of the SWPER indicator and its validity can be found in other literature [38]. Three broad domains have been established to assess women’s empowerment: attitude toward violence, social independence, and decision‐making authority/roles. These three dimensions were articulated by Ewerling et al. [38] in a manner akin to that of Miedema et al. [39] to represent enabling preconditions, instrumental agency, and intrinsic agency for women. Enabling preconditions refer to the factors that empower a woman, such as education and age. Instrumental agency pertains to a woman’s capacity to make decisions within the household, while intrinsic agency is connected to her attitudes and beliefs about gender norms, including the acceptability of spousal violence. Principal component analysis was employed to construct three domains based on 14 DHS questions. To ensure global relevance and comparability, we standardized the scores according to the global mean for LMICs and the standard deviation (SD) derived from a prior study [38]. Positive values of the standardized scores indicate above‐average empowerment, and negative values indicate below‐average empowerment, with zero signifying the mean value for LMICs [34, 38]. The single hot‐deck imputation technique was utilized to estimate the age of first childbirth for women without a history of giving birth.

### 2.4. Control Variables

The control variables in this analysis comprise current age categories (15–24, 25–34, and 35–49 years), religious affiliation (non‐Muslims and Muslims), number of living children (0–1, 2, and ≥3), current working status (yes or no), hormonal contraceptive usage (no or yes), wealth index classifications (poorest, poorer, middle, richer, and richest), place of residence (urban or rural), and regional distinctions. The DHS’s wealth index categorizes families into five quintiles from the poorest to the richest to quantify relative wealth within a country. The wealth index was created using principal component analysis to quantify household socioeconomic status by pooling household ownership of valued commodities.

### 2.5. Missing Data Analysis

Although listwise deletion, or complete case analysis, is the predominant strategy for handling missing data, other researchers contend that this technique may introduce bias in parameter estimates. The exclusion of individuals with missing data may have led to selection bias if the missingness was nonrandom, resulting in distorted estimates of the association and a lack of external validity, which refers to the generalizability of the study results to the intended population [40]. However, if the assumption of missing completely at random (MCAR) is valid, utilizing listwise deletion is acknowledged to produce unbiased estimates and conservative outcomes. When the data do not conform to the assumption that they are MCAR, utilizing listwise deletion may induce a bias in the parameter estimates [41]. However, when there is a sufficiently large sample, adequate power, and the assumption that the data are MCAR holds true, using listwise deletion may be a justifiable strategy [42]. Therefore, we employed Little’s MCAR test to ascertain that the missing data were MCAR, and the analysis did not support MCAR (*p*  < 0.001). Chi‐square tests indicated notable differences between included and excluded participants: excluded women were more frequently in the younger age group of 15–24 years (25.7% vs. 75.1% missing, *p*  < 0.001) and belonged to the lowest income households (17.6% vs. 28.9% missing, *p*  < 0.001). The observed patterns indicate that the data likely exhibit missing at random (MAR) in relation to the observed covariates, rather than being MCAR. Although multiple imputation is frequently suggested, complete case analysis can yield valid estimates under MAR assumptions when the missing data are associated with the observed variables incorporated in the analysis model [43]. Nevertheless, the sensitivity analysis revealed that the results remained consistent irrespective of the application of multiple imputation or listwise deletion to handle the missing data.

### 2.6. Statistical Analysis

Using Stata 14.2 (College Station, TX: StataCorp LLC), univariate descriptive analyses were performed to assess the completeness and distributions of all variables. The outcome variables were evaluated both continuously and dichotomously. Consequently, the bivariate association between the women’s empowerment score and the GAD‐7 and PHQ‐9 scores was evaluated by computing Pearson correlation coefficients. We employed one‐way analysis of variance (ANOVA) or *ꭓ* ^2^ tests, as applicable, to evaluate the bivariate relationship between various sociodemographic characteristics and GAD‐7 and PHQ‐9 scores, as well as the presence of moderate to severe anxiety and depressive symptoms. ANOVA was also conducted to evaluate the association between the empowerment scores and various sociodemographic variables. Tukey’s honestly significant difference (HSD) post hoc multiple comparison tests were utilized as follow‐ups to a significant ANOVA when applicable. Multivariable linear regression models were utilized to assess the relationships between various aspects of women’s empowerment scores and the GAD‐7 and PHQ‐9 scores. A directed acyclic graph (DAG) was constructed (Figure 1) to represent our data analysis strategy (multivariable analyses). Confounders, mediators, and colliders were identified based on existing knowledge regarding the relationship between exposure and outcome [25, 44–49]. We adjusted for confounders; however, we did not adjust for mediators, as our focus is on estimating the net association of the exposure and the outcome. When mediating variables are accounted for in the model, the path will be closed, resulting in the absence of the net relationship of exposure to the outcome. Furthermore, this may lead to an over‐adjustment bias in the estimation of the net association, thereby diminishing precision [50]. The DAG (Figure 1) identifies, based on previous literature [20–23, 26–28], age, religion, wealth index, working status, place of residence, and regional distinction as confounders for estimating the net relationship of women’s empowerment with the GAD‐7 or PHQ‐9 score, which pertains to mental health issues. The literature indicates that empowering women leads to an increase in contraception utilization and a decrease in the number of children [51–54]. Conversely, an increased number of children and the utilization of contraception are associated with an increased likelihood of mental health issues among women [55–57]. Therefore, these factors serve as intermediate mechanisms through which women’s empowerment is linked to mental health issues (Figure 1). All SWPER domains are incorporated into the regression models simultaneously with confounding variables, and the adjusted regression coefficients along with their related standard errors are reported herein. Sensitivity analysis was performed, incorporating each domain individually into the model, and the results were consistent with those obtained when all domains were included simultaneously in the model (Table S1). We further tested for interactions between each domain of empowerment and confounding variables, such as age, wealth index, place of residence, and region, to see whether the associations between women’s empowerment and mental health issues were modified by these confounding variables.

**Figure 1:**
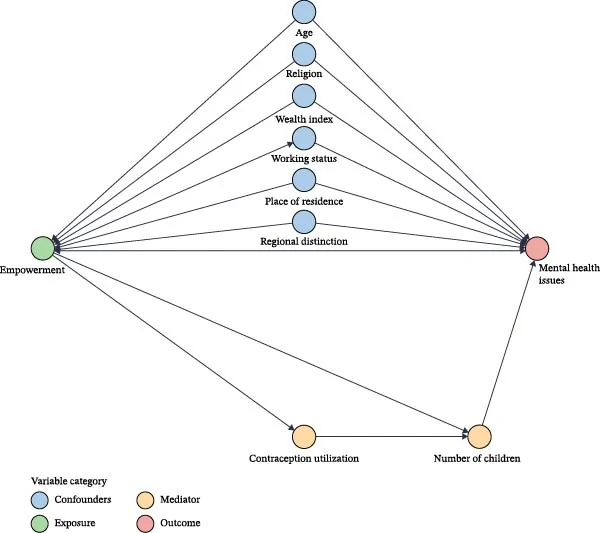
Directed acyclic graph for the association between women’s empowerment (SWPER index) and mental health issues.

The secondary multivariable analysis estimated the association between women’s empowerment scores and the presence of moderate to severe anxiety and depression symptoms using multivariable logistic regression while adjusting for the selected covariates. The findings were articulated through odds ratios (ORs) accompanied by their respective 95% confidence intervals (CIs). We determined the events per predictor parameter by dividing the number of events utilized in model development by the number of candidate predictor parameters (number of degrees of freedom linked to the candidate predictors). The study involved 18 candidate predictors, yielding an event count per variable of 37.5 (675/18) for moderate to severe anxiety symptoms and 42.7 (769/18) for moderate to severe depression symptoms, both exceeding the recommended minimum of 10 events per variable [58]. The model’s goodness of fit was assessed using the Archer–Lemeshow test, an adaptation of the Hosmer‐Lemeshow test applicable to survey data. The Archer–Lemeshow test results, with *p*‐values of 0.905 for the moderate‐to‐severe anxiety model and 0.915 for the moderate‐to‐severe depression model, suggest that the mental health issues are not significantly different from those predicted by the model, thus demonstrating a good overall model fit. We confirmed the absence of multicollinearity to satisfy the regression assumption by employing the variance inflation factor. The *svy* commands in Stata were employed to accommodate the survey design of the Bangladesh DHS, incorporating PSUs, sampling weights, and stratification. Estimation commands utilizing the *svy* commands compute standard errors based on the Taylor linearization estimator by default. The *p*‐values were calculated as two‐sided, with statistical significance established at *p* = 0.05.

## 3. Results

### 3.1. Sample Characteristics

The majority of the respondents were Muslims (90.6%), and one‐third of the respondents (35.4%) had 3 or more living children. One in three respondents (33.2%) was found to be currently working; 42.9% used hormonal contraception, and more than half of the respondents were from rural areas (Table 1). The prevalence of moderate to severe anxiety (GAD‐7 ≥ 10) and depression (PHQ‐9 ≥ 10) symptoms in this sample was found to be 4.2% and 4.7%, respectively (Figure 2). The study also found that the average scores of the women’s empowerment dimensions were 0.57 (SD 0.48), −0.38 (SD 0.74), and 0.25 (SD 0.90) for attitude toward violence, social independence, and decision‐making, respectively (Table 2).

**Figure 2:**
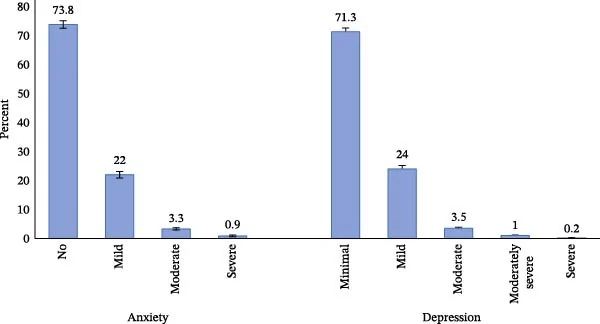
Symptoms of anxiety and depression at different thresholds.

**Table 1:** Demographic characteristics of nationally representative samples of women in Bangladesh aged 15–49 years (*n* = 15,813).

Characteristics	Number^a^	Percent^b^	95% CI
Age			
15–24	3230	21.0	20.2–21.8
25–34	5841	36.9	35.9–37.9
35–49	6742	42.1	41.2–43.1
Religion			
Non‐Muslims	1587	9.4	7.7–11.4
Muslims	14,226	90.6	88.5–92.2
No. of living children			
0–1	3966	25.3	24.4–26.1
2	6234	39.3	38.3–40.3
≥3	5613	35.4	34.3–36.5
Wealth index			
Poorest	2979	18.7	17.4–20.0
Poorer	3207	20.7	19.7–21.7
Middle	3184	20.9	19.9–21.9
Richer	3252	20.7	19.6–21.9
Richest	3191	18.9	17.4–20.5
Currently working			
No	10,747	66.8	65.1–68.4
Yes	5066	33.2	31.6–34.8
Hormonal contraceptive use			
No	8997	57.1	55.8–58.3
Yes	6816	42.9	41.7–44.1
Place of residence			
Urban	5350	27.4	26.1–28.6
Rural	10,463	72.6	71.3–73.8
Region			
Barishal	1690	6.0	5.6–6.4
Chattogram	2364	18.7	17.6–19.8
Dhaka	2361	24.8	23.6–25.9
Khulna	2104	12.2	11.6–12.8
Mymensingh	1725	7.7	7.3–8.0
Rajshahi	2049	13.4	12.5–14.2
Rangpur	1970	11.9	11.3–12.3
Sylhet	1550	5.3	5.0–5.5

Abbreviation: CI, confidence interval.

^a^Numbers are unweighted.

^b^In estimating percentages, the complex survey design and sampling weights are taken into account.

**Table 2:** Summary statistics of SWPER women’s empowerment dimensions.

Statistics	Attitude toward violence	Social independence	Decision‐making
Mean	0.57	−0.38	0.25
Standard deviation	0.48	0.74	0.90
25^th^ percentile	0.67	−0.88	−0.61
Median	0.72	−0.42	0.82
75^th^ percentile	0.75	0.05	0.92
Minimum	−2.45	−2.69	−2.10
Maximum	0.93	4.08	1.26

### 3.2. Association Between Women’s Empowerment and Anxiety and Depression Symptoms

The attitude toward violence and the social independence domain were found to be significantly negatively associated with GAD‐7 (Pearson correlation coefficient [*r*]: −0.077) and PHQ‐9 (*r*: −0.053) scores. On the other hand, the decision‐making domain was positively associated with GAD‐7 (*r*: 0.036) and PHQ‐9 (*r*: 0.020) scores (Table 3). The mean GAD‐7 and PHQ‐9 scores were higher among older women (aged 35–49 years), Muslims, those with three or more children, and currently working women.

**Table 3:** Demographic characteristics of nationally representative samples of women in Bangladesh aged 15–49 years and association with moderate to severe anxiety and depression symptoms.

Characteristics	Mean (SD) or No (%)^a^		*p*‐Value (*F*/*ꭓ* ^2^‐test)	Mean (SD) or No (%)^a^		*p*‐Value (*F*/*ꭓ* ^2^‐test)
	GAD‐7 score	Moderate to severe anxiety (*n* = 675)		PHQ‐9 score	Moderate to severe depression (*n* = 769)	
Attitudes toward violence	−0.077^∗∗∗^^b^	0.48 (0.60)	<0.001	−0.053^∗∗∗^^b^	0.49 (0.57)	<0.001
Social independence	−0.080^∗∗∗^^b^	−0.50 (0.75)	<0.001	−0.53^∗∗∗^^b^	−0.50 (0.71)	<0.001
Decision‐making	0.036^∗∗∗^^b^	0.36 (0.83)	0.002	0.020^∗^^b^	0.33 (0.86)	0.016
Age	—	—	<0.001	—	—	<0.001
15–24	2.41 (2.75)	84 (2.5)	—	2.86 (3.11)	110 (3.2)	—
25–34	3.03 (3.07)	231 (3.8)	—	3.27 (3.25)	269 (4.6)	—
35–49	3.51 (3.32)	360 (5.4)	—	3.66 (3.48)	390 (5.6)	—
Religion	—	—	0.006	—	—	0.031
Non‐Muslims	2.67 (2.76)	42 (2.6)	—	2.98 (3.04)	54 (3.4)	—
Muslims	3.15 (3.18)	633 (4.4)	—	3.39 (3.37)	715 (4.9)	—
No. of living children	—	—	<0.001	—	—	<0.001
0–1	2.59 (2.83)	123 (2.9)	—	3.03 (3.17)	158 (4.0)	—
2	3.01 (3.06)	238 (3.8)	—	3.28 (3.27)	279 (4.2)	—
≥3	3.59 (3.37)	314 (5.6)	—	3.65 (3.50)	332 (5.8)	—
Wealth index	—	—	0.213	—	—	0.097
Poorest	3.38 (3.20)	146 (4.9)	—	3.47 (3.37)	162 (5.1)	—
Poorer	3.28 (3.18)	130 (4.0)	—	3.48 (3.36)	163 (5.0)	—
Middle	3.12 (3.15)	132 (4.2)	—	3.35 (3.38)	173 (5.3)	—
Richer	3.10 (3.22)	158 (4.5)	—	3.38 (3.39)	149 (4.4)	—
Richest	2.69 (2.91)	109 (3.4)	—	3.08 (3.16)	122 (3.7)	—
Currently working	—	—	0.889	—	—	0.828
No	3.07 (3.14)	450 (4.2)	—	3.31 (3.35)	522 (4.8)	—
Yes	3.19 (3.14)	225 (4.2)	—	3.44 (3.31)	247 (4.7)	—
Hormonal contraceptive use	—	—	0.040	—	—	0.932
No	3.18 (3.20)	411 (4.5)	—	3.42 (3.34)	442 (4.7)	—
Yes	3.01 (3.06)	264 (3.8)	—	3.25 (3.33)	327 (4.7)	—
Place of residence	—	—	0.263	—	—	0.088
Urban	3.04 (3.04)	217 (3.8)	—	3.35 (3.26)	236 (4.0)	—
Rural	3.14 (3.19)	458 (4.3)	—	3.35 (3.38)	533 (5.0)	—
Region	—	—	0.004	—	—	0.001
Barishal	3.23 (2.89)	65 (4.2)	—	3.40 (3.05)	76 (4.8)	—
Chattogram	3.26 (3.28)	116 (4.8)	—	3.52 (3.42)	97 (4.2)	—
Dhaka	2.80 (2.86)	81 (3.3)	—	3.07 (3.06)	93 (3.7)	—
Khulna	3.13 (3.42)	125 (5.6)	—	3.58 (3.66)	142 (6.4)	—
Mymensingh	2.67 (2.95)	51 (3.0)	—	3.86 (3.17)	62 (3.6)	—
Rajshahi	3.00 (2.86)	55 (2.8)	—	3.24 (3.10)	66 (3.4)	—
Rangpur	3.43 (3.30)	104 (5.6)	—	3.68 (3.56)	140 (7.6)	—
Sylhet	3.36 (3.44)	78 (4.8)	—	3.43 (3.56)	93 (5.9)	—
Total	3.11 (3.14)	675 (4.2)	—	3.35 (3.34)	769 (4.7)	—

^a^Numbers are unweighted; in estimating percentages, the complex survey design and sampling weights are taken into account.

^b^Pearson correlation coefficient.

^∗^ *p*  < 0.05.

^∗∗∗^ *p*  < 0.001.

Table 3 also presents the associations between SWPER scores and moderate‐to‐severe anxiety and depression. Women aged 35–49 (anxiety: 5.4% vs. 2.5%, *p*  < 0.001; depression: 5.6% vs. 3.2%, *p*  < 0.001), Muslims (anxiety: 4.4% vs. 2.6%, *p* = 0.006; 4.9% vs. 3.4%, *p* = 0.031), and those with three or more living children (anxiety: 5.6% vs. 2.9%, *p*  < 0.001; depression: 5.8% vs. 4.0%, *p*  < 0.001) were much more likely to have moderate to severe anxiety and depressive symptoms than women aged 15–24 years, non‐Muslims, and women with 0–1 living children (Table 3).

Figure 3 shows the unadjusted relationship between the SWPER score and the GAD‐7 and PHQ‐9 scores. The mean GAD‐7 and PHQ‐9 scores among women decreased linearly with increases in the SD of the attitude toward violence and social independence scores. However, the mean GAD‐7 and PHQ‐9 scores were found to increase linearly with an increased SD in the decision‐making domain.

**Figure 3:**
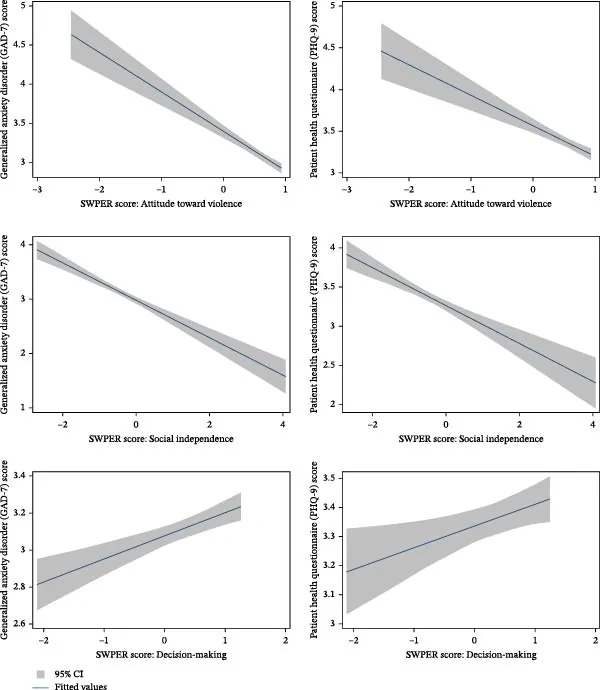
Relationship between women’s empowerment and anxiety (GAD‐7) and depression (PHQ‐9) scores.

### 3.3. Association Between SWPER Scores and Different Sociodemographic Variables

As displayed in Table 4, the mean empowerment score in relation to attitude toward violence was significantly higher among women aged 15–24 years (mean [SD]: 0.60 [0.43]), non‐Muslims (0.62 [0.37]), those with 0–1 living child (0.60 [0.43]), those from the richest households (0.65 [0.36]), and those from urban places of residence (0.61 [0.41]) than their respective counterparts. The mean score in relation to social independence was also significantly higher among women aged 25–34 years (−0.33 [−0.24]), non‐Muslims (−0.22 [0.79]), those with a low number of living children (−0.07 [0.74]), those from the richest households (−0.01 [0.93]), and those living in urban areas (−0.23 [0.83]). However, the mean score for decision‐making was higher among older women aged 35–49 years, those with two living children, women from the richest households, women who were currently working, those not using hormonal contraception, and women living in rural areas.

**Table 4:** Women empowerment (SWPER) scores by various sociodemographic characteristics.

Statistics	Women empowerment (SWPER) score					
	Attitude toward violence		Social independence		Decision‐making	
	M (SD)	*p*‐Value (*F*‐test)	M (SD)	*p*‐Value (*F*‐test)	M (SD)	*p*‐Value (*F*‐test)
Age	—	<0.001	—	<0.001	—	<0.001
15–24	0.60 (0.43)	—	−0.33 (0.56)	—	0.03 (0.99)	—
25–34	0.58 (0.47)	—	−0.24 (0.73)	—	0.29 (0.88)	—
35–49	0.53 (0.50)	—	−0.51 (0.78)	—	0.32 (0.85)	—
Religion	—	<0.001	—	<0.001	—	0.151
Non‐Muslims	0.62 (0.37)	—	−0.22 (0.79)	—	0.28 (0.87)	—
Muslims	0.56 (0.49)	—	−0.39 (0.72)	—	0.25 (0.90)	—
No. of living children	—	<0.001	—	<0.001	—	<0.001
0–1	0.60 (0.43)	—	−0.07 (0.74)	—	0.14 (0.95)	—
2	0.58 (0.46)	—	−0.32 (0.73)	—	0.29 (0.87)	—
≥3	0.52 (0.52)	—	−0.65 (0.62)	—	0.28 (0.88)	—
Wealth index	—	<0.001	—	<0.001	—	<0.001
Poorest	0.49 (0.57)	—	−0.57 (0.59)	—	0.18 (0.92)	—
Poorer	0.54 (0.51)	—	−0.49 (0.61)	—	0.21 (0.92)	—
Middle	0.56 (0.46)	—	−0.45 (0.65)	—	0.25 (0.90)	—
Richer	0.58 (0.45)	—	−0.36 (0.69)	—	0.26 (0.89)	—
Richest	0.65 (0.36)	—	−0.01 (0.93)	—	0.35 (0.85)	—
Currently working	—	0.002	—	<0.001	—	<0.001
No	0.57 (0.46)	—	−0.34 (0.71)	—	0.18 (0.93)	—
Yes	0.55 (0.50)	—	−0.44 (0.78)	—	0.39 (0.81)	—
Hormonal contraceptive use	—	0.956	—	0.001	—	0.012
No	0.56 (0.48)	—	−0.35 (0.79)	—	0.27 (0.89)	—
Yes	0.56 (0.47)	—	−0.40 (0.65)	—	0.23 (0.91)	—
Place of residence	—	<0.001	—	<0.001	—	<0.001
Urban	0.61 (0.41)	—	−0.23 (0.83)	—	0.30 (0.88)	—
Rural	0.54 (50)	—	−0.44 (0.66)	—	0.23 (0.91)	—
*p*‐Value						
Region	—	<0.001	—	<0.001	—	<0.001
Barishal	0.60 (0.42)	—	−0.34 (0.69)	—	0.15 (0.93)	—
Chattogram	0.57 (0.46)	—	−0.29 (0.65)	—	0.35 (0.85)	—
Dhaka	0.54 (0.50)	—	−0.30 (0.80)	—	0.29 (0.85)	—
Khulna	0.53 (0.54)	—	−0.44 (0.74)	—	0.17 (0.95)	—
Mymensingh	0.57 (0.49)	—	−0.39 (0.73)	—	0.27 (0.90)	—
Rajshahi	0.55 (0.49)	—	−0.47 (0.74)	—	0.32 (0.85)	—
Rangpur	0.60 (0.40)	—	−0.48 (0.75)	—	0.36 (0.84)	—
Sylhet	0.58 (0.45)	—	−0.27 (0.70)	—	0.01 (0.98)	—

### 3.4. Multivariate Results

Table 5 displays the relationships between empowerment domains, sociodemographic characteristics, and the GAD‐7 and PHQ‐9 scores. An increase of one unit in the SD of the attitude toward violence and social independence score correlated with a reduction of 0.34 units (*β*‐coefficient: −0.34) and 0.16 units (*β*‐coefficient: −0.16) in the adjusted scores on the GAD‐7, respectively. The PHQ‐9 score exhibited a reduction of 0.24 units (*β*‐coefficients: −0.24) and 0.13 units (*β*‐coefficients: −0.13) corresponding to each SD increase in attitudes toward violence and social independence, respectively. However, an additional SD in the decision‐making domain was associated with a 0.08‐unit increase in the GAD‐7 score. Table 5 also presents the odds of moderate to severe anxiety and depression symptoms among currently married women aged 15–49 years. A unit increase in the SD of the attitude toward violence decreases the odds of moderate to severe anxiety symptoms by 21% (OR: 0.79; 95% CI: 0.68–0.91) and depression symptoms by 20% (OR: 0.80; 95% CI: 0.69–0.94). However, one additional SD in the social independence domain decreases the odds of moderate to severe anxiety symptoms by 16% (OR: 0.84; 95% CI: 0.71–0.99) and depressive symptoms by 14% (OR: 0.86; 95% CI: 0.75–0.99). The secondary analyses yield results that align with the primary analyses, indicating the robustness of our findings.

**Table 5:** Results of multivariable analyses of the relationships between women’s empowerment and mental health issues among currently married women aged 15–49 years in Bangladesh.

Characteristics	GAD‐7 score	Moderate to severe anxiety		PHQ‐9 score	Moderate to severe depression	
	*β* (SE)	OR	95% CI	*β* (SE)	OR	95% CI
Attitudes toward violence	−0.34 (0.06) ^∗∗∗^	0.79^∗∗^	0.68–0.91	−0.24 (0.07) ^∗∗∗^	0.80^∗∗^	0.69–0.94
Social independence	−0.16 (0.04) ^∗∗∗^	0.84^∗^	0.71–0.99	−0.13 (0.04) ^∗∗^	0.86^∗^	0.75–0.99
Decision‐making	0.08 (0.04) ^∗^	1.08	0.95–1.23	0.03 (0.04)	1.05	0.93–1.18
Age						
15–24	—	1	—	—	1	—
25–34	0.69 (0.07) ^∗∗∗^	1.59^∗∗^	1.21–2.10	0.49 (0.08) ^∗∗∗^	1.49^∗∗^	1.16–1.91
35–49	1.13 (0.07) ^∗∗∗^	2.22^∗∗∗^	1.69–2.92	0.84 (0.08) ^∗∗∗^	1.78^∗∗∗^	1.42–2.22
Religion						
Non‐Muslims	—	1	—	—	1	—
Muslims	0.61 (0.12) ^∗∗∗^	1.89^∗∗^	1.29–2.77	0.51 (0.14) ^∗∗∗^	1.57^∗∗^	1.13–2.19
Wealth index						
Poorest	—	1	—	—	1	—
Poorer	−0.10 (0.10)	0.80	0.60–1.07	−0.004 (0.10)	1.02	0.78–1.34
Middle	−0.24 (0.11) ^∗^	0.88	0.65–1.19	−0.08 (0.12)	1.13	0.85–1.49
Richer	−0.33 (0.11) ^∗∗^	0.96	0.72–1.28	−0.14 (0.12)	0.93	0.69–1.24
Richest	−0.64 (0.12) ^∗∗∗^	0.74	0.52–1.04	−0.37 (0.13) ^∗∗^	0.82	0.58–1.14
Currently working						
No	—	1	—	—	1	—
Yes	−0.10 (0.07)	0.91	0.74–1.11	−0.03 (0.08)	0.86	0.70–1.04
Place of residence						
Urban	—	1	—	—	1	—
Rural	−0.10 (0.11)	0.99	0.76–1.31	0.09 (0.12)	1.04	0.79–1.36
Region						
Barishal	—	1	—	—	1	—
Chattogram	0.13 (0.21)	1.23	0.74–2.04	0.14 (0.21)	0.92	0.58–1.48
Dhaka	−0.35 (0.16) ^∗^	0.81	0.53–1.24	−0.35 (0.15) ^∗^	0.82	0.55–1.23
Khulna	−0.11 (0.18)	1.35	0.89–2.06	0.09 (0.18)	1.39	0.96–2.02
Mymensingh	−0.58 (0.18) ^∗∗^	0.66	0.42–1.04	−0.58 (0.19) ^∗∗^	0.73	0.49–1.08
Rajshahi	−0.28 (0.19)	0.62	0.38–1.04	−0.25 (0.17)	0.66^∗^	0.44–0.99
Rangpur	0.31 (0.18)	1.37	0.90–2.08	0.41 (0.18) ^∗^	1.68^∗∗^	1.14–2.46
Sylhet	0.16 (0.21)	1.16	0.74–1.82	0.04 (0.23)	1.31	0.84–2.05

Abbreviations: 95% CI, 95% confidence interval; GAD, generalized anxiety disorder; OR, odds ratio; PHQ, Patient Health Questionnaire.

^∗^ *p*  < 0.05.

^∗∗^ *p*  < 0.01.

^∗∗∗^ *p*  < 0.001.

Although we tested several interaction terms, this study only identified noteworthy interactions between the social independence domain and place of residence (Table S2). The interaction term between SWPER domains and rural residence yielded *p* = 0.002 for the GAD‐7 score and *p* = 0.013 for the PHQ‐9 score in the social independence domain and *p* = 0.014 for the PHQ‐9 score in the decision‐making domain. This interaction implies that initiatives aimed at fostering gender‐specific development and enhancing women’s social independence, such as education and delayed marriage, and enhancing women’s participation in household decisions, could positively influence the mental health of women residing in rural areas.

## 4. Discussion

This research investigates the relationship between women’s empowerment and mental health issues among currently married women of reproductive age (15–49 years), employing nationally representative data. This study broadens the exploration of women’s mental health and empowerment by investigating the potential correlations between various dimensions of empowerment and the presence of anxiety and depression symptoms. This sample revealed that the prevalence of moderate‐to‐severe anxiety and depression symptoms was 4.2% and 4.7%, respectively. The findings further indicated a link between mental health issues and various dimensions of women’s empowerment. The presence of anxiety and depression symptoms exhibited an inverse correlation with attitudes toward violence and the social independence aspect of women’s empowerment. The results of this research highlight the importance of empowering women for their mental well‐being.

This research indicates that specific dimensions of women’s empowerment are associated with mental health issues, aligning with the body of literature globally [20–25]. Our findings indicate that a higher SD score in women’s attitudes toward violence is inversely associated with anxiety and depressive symptoms. In numerous developing nations, such as Bangladesh, there exists a widespread acceptance of domestic violence against women, particularly in the form of wife‐beating. This phenomenon is frequently sustained by prevailing societal norms and established gender roles [59]. Consequently, many women in these societies deem such violence justifiable, acknowledging their husbands’ authority to regulate their actions, even through violent means [35, 60]. Previous research has established that women who rationalize any form of violence perpetrated by a spouse are less inclined to disclose such issues to legal authorities and are more susceptible to enduring psychological challenges [61, 62]. Furthermore, it has been observed that women with diminished self‐esteem who pursue validation from their partners yet encounter rejection and devaluation are especially prone to experiencing emotional turmoil and depressive symptoms [63]. The link between attitudes toward violence and adverse mental health outcomes could also be elucidated by the direct correlation observed between women’s acceptance of violence and the prevalence of violence against women [64–66]. Previous research has established that women’s exposure to violence serves as a significant risk factor for their mental health challenges, such as anxiety and depression [32, 67, 68].

The findings of our study indicated that women with increased SD scores in the social independence domain of empowerment demonstrated a reduced likelihood of exhibiting symptoms associated with anxiety and depression. The domain of social independence signifies a woman’s intellectual growth and standing, potentially linked to her understanding of the significance of mental well‐being and heightened awareness of mental health concerns. Furthermore, the domain of social independence encompasses elements such as educational attainment, access to information, the timing of significant life events like marriage or cohabitation, and spousal age differentials. These factors serve as essential prerequisites that enable women to realize their aspirations and may also be linked to mental health challenges [69]. For instance, the favorable correlation between education and health is quite logical; one can reasonably anticipate that increased education contributes to improved physical and mental health outcomes. A range of theoretical frameworks elucidate the connection between education and health outcomes, encompassing both physical and mental aspects. Fundamental Cause Theory [70] posits that education serves as a fundamental determinant of health, as it facilitates access to resources that promote well‐being, including increased income, superior health care, healthier food options, and an overall healthier lifestyle. Human Capital Theory [71] conceptualizes education as an investment that generates returns for the individual, encompassing enhanced abilities, skills, and resources, which can lead to improved health outcomes. Researchers have posited that education fosters enhanced personal agency [72] and diminishes propensity for risk‐taking [73], both of which correlate with improved physical and mental well‐being. Consequently, considering the parameters established for this domain, it is reasonable to assert that women who possess a comprehensive understanding and have not interrupted their education or careers due to early life circumstances such as marriage or childbirth are less susceptible to mental health challenges [74].

Increased decision‐making power has been documented in an earlier study as negatively associated with common mental disorders [21]; however, our findings reveal that a higher SD score in the decision‐making domain of empowerment is positively correlated with anxiety symptoms only. Although surprising, this observation aligns with a recent study conducted in Bangladesh and Nepal [25, 75]. Such an inconsistent finding requires further in‐depth research for a comprehensive understanding.

The findings of this study hold significant implications for enhancing the mental well‐being of women in societies, such as Bangladesh, where patriarchy is deeply entrenched. The cultural and traditional patriarchal social norms significantly shape the status of women in Bangladesh. In the context of a societal framework characterized by male predominance and patriarchal structures in Bangladesh, men exert control over women and establish the parameters of relationships through familial ties, marriage, and inheritance practices [76]. Historically, women in Bangladesh have predominantly occupied roles within the domestic sphere, engaging primarily in household responsibilities and the care of children. Conversely, men engage in external labor, provide financial support for the family, and exert control over capital resources. This conventional role undermines women’s ability to attain parity with men. In opposition to established legislative norms, such practices are accepted within societal frameworks as cultural norms, resulting in their continued existence. Considering the detrimental impact of oppressive systems and ideologies on the mental well‐being of those subjected to them [77], it is reasonable to assert that patriarchy, as a gender‐based hierarchical structure, is likely to have profound implications for mental health. Moreover, in Bangladesh, the phenomenon of child marriage is profoundly rooted in cultural traditions, economic hardship, and disparities in gender equality [78]. A girl born into a financially disadvantaged family and compelled into an early marriage is likely to face the cessation of her education, bear children at a young age, encounter complications during pregnancy and childbirth, and suffer from violence more frequently than a girl who marries later and resides in a household with greater economic stability [79]. Thus, the findings of this investigation regarding the negative relationship between specific domains of the SWPER index, attitudes toward violence and social independence, and the likelihood of experiencing anxiety and depressive symptoms hold important implications for enhancing the mental health status of reproductive‐aged women in Bangladesh.

When evaluating the findings of this study, it is important to acknowledge its limitations, specifically that it was a cross‐sectional survey, which precludes the ability to establish cause‐and‐effect relationships among the examined variables. Although our findings demonstrate significant relationships, the potential for reverse causation remains, as evidenced by other research indicating that underlying mental health difficulties may diminish women’s decision‐making authority and heighten susceptibility to intimate partner violence [80, 81]. To comprehensively elucidate the complex connections between women’s empowerment and mental health issues, subsequent longitudinal studies should evaluate the bidirectional and temporal dynamics between women’s empowerment and symptoms of anxiety or depression, employing repeated measures and rigorously controlling for previous mental health status and other potential confounding variables influencing both exposure and outcome. Second, this study utilized secondary data, limiting the analysis to the available variables. Consequently, our investigation was unable to consider various additional unmeasured confounding variables, including exposure to violence, marital relationships, prior mental health conditions, social support, or chronic illnesses, all of which may have a significant impact on the estimated associations between women’s empowerment and mental health issues. Third, the mental health issues of women, including anxiety and depressive symptoms, were evaluated using self‐reported measures, which may be subject to desirability bias. Furthermore, despite employing proven and commonly utilized screening instruments to assess mental health issues, the self‐reported symptoms may not correspond with clinical diagnoses. Fourth, empowerment is a multifaceted concept encompassing economic, sociological, familial, interpersonal, legal, political, societal, and psychosocial components. The SWPER index includes three categories of empowerment, although it is difficult to fully capture all facets of empowerment within a singular index. Furthermore, it is important to recognize that the relationship identified at the group level may not necessarily reflect an identical relationship at the individual level. Moreover, certain measures utilized in the SWPER, including attitudes toward violence, may be prone to social desirability bias, potentially resulting in an underestimation. Finally, the data analyzed in this study are exclusively quantitative, lacking any qualitative data to examine the perspectives of women’s empowerment among these individuals. Consequently, to attain a more profound comprehension of the association between empowerment and mental health, it would be beneficial to investigate the context‐specific social and cultural attributes of women using supplementary data obtained via qualitative approaches.

In conclusion, this study found an inverse relationship between women’s empowerment and their experience of mental health symptoms, indicating that higher levels of empowerment may be associated with better mental health. Therefore, national mental health policy in Bangladesh should prioritize empowering women for the advancement and enhancement of their mental well‐being. Empowerment initiatives should maintain their emphasis on improving educational outcomes for women, preventing early marriages and childbirth, and expanding access to information. Additionally, it is essential to tackle the fundamental factors influencing empowerment, including social norms and patriarchal systems that restrict women’s voices and safeguard those lacking agency. Future in‐depth and longitudinal studies incorporating various empowerment indicators and a broad spectrum of confounding variables are essential to ascertain the extent of the relationship between women’s empowerment and mental health, as well as the mechanisms by which empowerment mitigates mental health challenges in women, thereby enhancing our understanding of this complex relationship.

## Funding

This research did not receive any specific grant from funding agencies in the public, commercial, or not‐for‐profit sectors.

## Conflicts of Interest

The authors declare no conflicts of interest.

## Supporting Information

Additional supporting information can be found online in the Supporting Information section.

## Supporting information


**Supporting Information** This article includes Tables S1 and S2. Table S1 shows results from multivariable analyses of associations between women’s empowerment and mental health symptoms among currently married women aged 15–49 years in Bangladesh, with each empowerment domain analyzed separately. Table S2 presents results from regression analyses of interaction effects.

## Data Availability

The data that support the findings of this study are available from the DHS Program at https://dhsprogram.com/, Reference Number 180657.0.001.BD.DHS.02.
