# Supplementary material for: Women Empowerment and Its Relationship With Anxiety and Depression Symptoms Among Currently Married Women in Bangladesh: A Nationally Representative Cross‐Sectional Study
Source: Depress Anxiety. 2026 Oct 31;2026:7941286. doi: 10.1155/da/7941286 (PMC13723310; doi:10.1155/da/7941286)
Supplement: Supplementary file 1 [file DA-2026-7941286-s001.docx]

**Supplement Table 1** Results of multivariable analyses of the relationships between women’s empowerment and mental health issues among currently married women aged 15–49 years in Bangladesh.

| **Characteristics** | **GAD-7 score** | | | **PHQ-9 score** | | |
| --- | --- | --- | --- | --- | --- | --- |
|  | β (SE) | | | β (SE) | | |
| **Attitudes toward violence** | -0.35 (0.06)*** |  |  | -0.25 (0.07)*** |  |  |
| **Social independence** |  | -0.17 (0.04)*** |  |  | -0.14 (0.04)*** |  |
| **Decision-making** |  |  | 0.07 (0.04) |  |  | 0.02 (0.04) |
| **Age** |  |  |  |  |  |  |
| 15–24^®^ | - | - | - | - | - | - |
| 25–34 | 0.70 (0.07)*** | 0.73 (0.07)*** | 0.70 (0.07)*** | 0.48 (0.08)*** | 0.50 (0.08)*** | 0.49 (0.08)*** |
| 35–49 | 1.19 (0.07)*** | 1.18 (0.07)*** | 1.20 (0.07)*** | 0.87 (0.08)*** | 0.86 (0.07)*** | 0.89 (0.08)*** |
| **Religion** |  |  |  |  |  |  |
| Non-Muslims^®^ | - | - | - | - | - | - |
| Muslims | 0.63 (0.12)*** | 0.63 (0.12)*** | 0.65 (0.12)*** | 0.53 (0.14)*** | 0.52 (0.14)*** | 0.55 (0.14)*** |
| **Wealth Index** |  |  |  |  |  |  |
| Poorest^®^ | - | - | - | - | - | - |
| Poorer | -0.11 (0.10) | -0.11 (0.10) | -0.13 (0.10) | -0.01 (0.10) | -0.01 (0.10) | -0.03 (0.10) |
| Middle | -0.26 (0.11)* | -0.26 (0.11)* | -0.29 (0.11)** | -0.09 (0.12) | -0.09 (0.12) | -0.12 (0.12) |
| Richer | -0.35 (0.11)** | -0.35 (0.11)** | -0.40 (0.11)*** | -0.16 (0.12) | -0.16 (0.12) | -0.19 (0.12) |
| Richest | -0.71 (0.12)*** | -0.68 (0.12)*** | -0.79 (0.12)*** | -0.43 (0.13)** | -0.40 (0.13)** | -0.48 (0.13)*** |
| **Currently working** |  |  |  |  |  |  |
| No^®^ | - | - | - | - | - | - |
| Yes | -0.09 (0.07) | -0.09 (0.07) | -0.10 (0.07) | -0.02 (0.08) | -0.02 (0.08) | 0.03 (0.08) |
| **Place of residence** |  |  |  |  |  |  |
| Urban^®^ | - | - | - | - | - | - |
| Rural | -0.09 (0.11) | -0.09 (0.11) | -0.08 (0.11) | -0.08 (0.12) | -0.09 (0.12) | -0.08 (0.12) |
| **Region** |  |  |  |  |  |  |
| Barishal^®^ | - | - | - | - | - | - |
| Chattogram | 0.14 (0.21) | 0.16 (0.21) | 0.15 (0.21) | 0.15 (0.21) | 0.16 (0.21) | 0.16 (0.21) |
| Dhaka | -0.33 (0.16)* | -0.31 (0.16) | -0.30 (0.16) | -0.34 (0.15)* | -0.32 (0.15)* | -0.31 (0.15)* |
| Khulna | -0.09 (0.18) | -0.08 (0.18) | -0.05 (0.18) | 0.11 (0.18) | 0.11 (0.18) | 0.13 (0.18) |
| Mymensingh | -0.56 (0.18)** | -0.56 (0.18)** | -0.57 (0.18)** | -0.58 (0.18)** | -0.57 (0.19)** | -0.57 (0.19)** |
| Rajshahi | -0.24 (0.19) | -0.24 (0.19) | -0.22 (0.19) | -0.22 (0.17) | -0.23 (0.17) | -0.21 (0.17) |
| Rangpur | 0.35 (0.18) | 0.32 (0.18) | 0.33 (0.18) | 0.43 (0.18)* | 0.41 (0.18)* | 0.42 (0.18)* |
| Sylhet | 0.14 (0.21) | 0.16 (0.21) | 0.16 (0.21) | 0.03 (0.23) | 0.04 (0.23) | 0.04 (0.23) |

*Note:* SE, standard error**;** GAD, generalized anxiety disorder; PHQ, patient health questionnaire; *p<.05, **p<.01, ***p<.001.

**Supplement Table 2** Results of regression analysis with interaction effect

| **Characteristics** | **GAD-7 score** | | **PHQ-9 score** | |
| --- | --- | --- | --- | --- |
|  | β (SE) | p-value | Β (SE) | p-value |
| **Attitudes toward violence** | -0.17 (0.13) | 0.177 | -0.05 (0.14) | 0.705 |
| **Social independence** | -0.32 (0.06)*** | <0.001 | -0.27 (0.07)*** | <0.001 |
| **Decision-making** | 0.01 (0.07) | 0.900 | -0.12 (0.07) | 0.067 |
| **Place of residence** |  |  |  |  |
| Urban^®^ | - |  |  |  |
| Rural | 0.08 (0.15) | 0.573 | 0.06 (0.16) | 0.709 |
| **Att toward violence X place of residence** |  |  |  |  |
| Att to violence X rural | -0.22 (0.14) | 0.130 | -0.24 (0.15) | 0.128 |
| **Social independence X place of residence** |  |  |  |  |
| Soc independence X rural | 0.25 (0.08)** | 0.002 | 0.22 (0.09)* | 0.013 |
| **Decision making X place of residence** |  |  |  |  |
| Decision making X rural | 0.10 (0.08) | 0.231 | 0.20 (0.08)* | 0.014 |

*Note:* Models are adjusted with all other confounders; SE, standard error**;** GAD, generalized anxiety disorder; PHQ, patient health questionnaire; *p<.05, **p<.01, ***p<.001.
